# Early prediction of longitudinal treatment adherence in obstructive sleep apnea using machine learning approaches

**DOI:** 10.1186/s13040-026-00554-0

**Published:** 2026-04-15

**Authors:** Máximo Domínguez-Guerrero, Daniel Álvarez, Verónica Barroso-García, María Fernández-Vaquerizo, Tomás Ruiz-Albi, Roberto Hornero

**Affiliations:** 1 Biomedical Engineering Group, University of Valladolid, Valladolid, 47011 Spain; 2 Centro de Investigación Biomédica en Red en Bioingeniería, Biomateriales y Nanomedicina (CIBER-BBN), Valladolid, 47001 Spain; 3 Pneumology Department, Río Hortega University Hospital, Valladolid, 47012 Spain

**Keywords:** Obstructive sleep apnea, CPAP adherence, Machine learning, Multilayer perceptron, Support vector machines, Random forest

## Abstract

**Background:**

Continuous Positive Airway Pressure (CPAP) is the most prescribed treatment for Obstructive Sleep Apnea (OSA), but adherence remains a critical challenge, especially long term. This study aims to predict CPAP adherence at 3, 6, and 12 months based on baseline patient status and usage data from initial 30 days of treatment.

**Results:**

A retrospective cohort of 2180 patients was analyzed. Feature selection was performed using the maximum relevance minimum redundancy (mRMR) algorithm with bootstrapping. Three machine learning (ML) models were trained and evaluated: support vector machine (SVM), random forest (RF), and multilayer perceptron (MLP). A core subset of early CPAP usage variables (median nightly use, days of use, and 30-day adherence) emerged as the most relevant features across all timepoints. Each prediction window also revealed exclusive features, suggesting that adherence at different stages may be driven by distinct factors. At 3 months, MLP exhibited the strongest predictive capacity (kappa = 0.823, AUC = 0.910). At 6 months, RF and SVM models yielded the highest results (kappa = 0.727, AUC = 0.863), whereas at 12 months, RF consistently outperformed the other algorithms (kappa = 0.698, AUC = 0.849), underscoring its robustness in forecasting long-term adherence.

**Conclusions:**

Our results suggest that ML algorithms can effectively predict CPAP adherence using very short-term usage data. Accordingly, our models are able to provide efficient early identification of patients at risk of non-adherence in order to support personalized interventions, improve outcomes, and reduce the healthcare burden in OSA management.

## Background

Obstructive sleep apnea (OSA) is a chronic condition characterized by recurrent episodes of partial or total obstruction of the upper airway during sleep, leading to reduced or absent airflow despite continued respiratory effort [1]. This disease is one of the most prevalent sleep disorders worldwide. It is estimated that the global prevalence varies between 4% and 30% [1, 2].

OSA has been associated with a wide range of adverse consequences, including cardiovascular and cerebrovascular complications, metabolic and neurodegenerative disorders, increased risk of traffic and occupational accidents and reduced quality of life [1]. These consequences can be even more severe if the disease is not effectively treated [3].

The most thoroughly researched technique for non-invasive respiratory support is continuous positive airway pressure (CPAP) device, which deliver continuous pressurized air to the upper airway to prevent its collapse during sleep [4]. It is favored over other options like surgery, transcutaneous electrical stimulation, and medication, as these are generally more invasive and less effective in most cases [5]. The therapeutic benefits of CPAP are directly linked to the duration of nightly use, making consistent adherence essential for effective treatment. However, a significant proportion of patients report discomforts associated with CPAP use, including nasal irritation, dry throat and mouth, claustrophobia, skin creases, irritation, and air leakage [6]. These adverse effects often lead patients to reduce or discontinue treatment altogether. CPAP non-adherence has serious consequences, not only diminishing patient quality of life but also increasing the burden on healthcare systems [7].

It is estimated that up to 20% of patients discontinue CPAP within the first year, and only 68% remain adherent beyond the fourth month of treatment [8]. The absence of reliable predictive models for CPAP adherence, together with the uncertainty about the most influential factors, underscores a critical gap in current clinical knowledge.

Previous studies have addressed the challenge of predicting CPAP adherence using various machine learning (ML) approaches and clinical variables [9–12]. While ML represents the current state of the art in this field, no predictive model has consistently outperformed others across studies. Reported performance has generally been modest, with AUC values ranging from approximately 0.70 to 0.76 across different cohorts and prediction windows [10–12]. The literature also reveals substantial variability in the temporal windows of prediction, hindering direct comparisons between studies. Furthermore, many risk factors potentially influencing CPAP adherence remain poorly understood, which continues to limit the development of early, generalizable, and reliable predictive tools.

Other studies have also explored this problem, aiming to identify relevant factors associated with CPAP adherence and subsequently build predictive models [7, 13–16]. However, differences in methodology, feature sets, and adherence definitions are difficult for fair comparison across studies.

Our hypothesis is based on the premise that adherence to CPAP therapy can be accurately predicted in the short, medium, and long term using an appropriate combination of clinical and usage variables as inputs to a machine learning model. To test this hypothesis, it is well established that the first essential step is to identify the key factors that influence adherence outcomes. In this study, the minimum redundancy maximum relevance (mRMR) algorithm is employed to identify an optimized subset of predictive features. Subsequently, three well-established ML algorithms (Support Vector Machine (SVM) [17], Random Forest (RF) [18], and Multilayer Perceptron (MLP) [19]) are utilized to model and predict treatment adherence at three clinically relevant time points: 3, 6, and 12 months.

The main novelties of this study are the simultaneous comparison of multiple ML algorithms across three prediction windows, and the integration of a robust feature selection strategy based on mRMR algorithm with bootstrap resampling. This dual approach not only identifies the most relevant predictors of CPAP adherence at different stages but also highlights the dynamic nature of adherence determinants over time.

The objectives of this study are: (1) to identify the most influential factors associated with CPAP adherence at short, medium, and long term; and (2) to design, train, and validate predictive clinically useful tools.

## Methods

A retrospective observational study of predictive model design and validation was conducted in the Pulmonology Department of the Río Hortega University Hospital (RHUH) in Valladolid, Spain. According to standard clinical protocols, patients with severe OSA, or moderate OSA with symptoms, were prescribed with home CPAP (AirSense 10, ResMed). Usage data were collected from the MyOSA platform (Oxigen Salud S.A.), which connects CPAP devices with a centralized database designed to store usage variables, allowing pulmonologists to follow-up treatment adherence.

All patients were continuously telemonitored for 30 days (one record per day), and to account for the dynamic nature of treatment engagement, adherence was evaluated independently at 3, 6, and 12 months. Furthermore, patients who discontinued treatment or abandoned telemonitoring at any follow-up point were categorized as “non-adherent”. For patients remaining in the program, adherence status was re-assessed at each milestone based on current usage metrics. This approach was adopted to minimize selection bias and ensure the results accurately reflect real-world clinical outcomes.

The adherence criteria, based on the guidelines of the American Thoracic Society (ATS) [20], was defined as usage greater than or equal to 4 h per night on at least 70% of nights during the monitoring period under analysis.

For each patient, we collected anthropometric, sociodemographic, comorbidity information, and common respiratory disturbance indexes from the patients’ diagnostic polysomnography (PSG). Furthermore, the dataset includes records of CPAP usage during the first 30 days of treatment, including daily hours of use, percentage of nights using the device, percentage of nights using the device more than 4 h, mask leakage, obstructive events, and month of treatment initiation. The longitudinal follow-up milestones (3, 6, and 12 months) were calculated starting from the date of CPAP treatment initiation. Initial usage features were derived from the first 30 days of this treatment period, including the binary 30-day adherence status calculated according to ATS guidelines. At each follow-up, the number of non-adherent and adherent patients were 947–1233, 970–1210, and 983–1197, respectively.

Possible outliers were identified using the explainable OUTLIERTREE algorithm [21], which utilizes decision tree conditioning to detect anomalous observations based on conditional distributions. This procedure identified 4.3% of the cohort as potential outliers. However, these patients were retained in the final analysis as they represented clinically plausible profiles and their inclusion did not affect the overall stability of the models, as confirmed by a sensitivity analysis.

The study protocol was approved by the Ethics Committee for clinical research of the RHUH (REF: PI-24-433-O). All patients were informed and provided written consent for the collection and processing of their clinical and CPAP adherence data. This research was conducted in accordance with the ethical principles outlined in the Declaration of Helsinki and complies with current data protection laws and patient rights legislation.

Tables 1, 2 and 3 summarize baseline characteristics, indexes derived from the diagnostic PSG, and usage variables from the 30-day follow-up, respectively.

The full dataset was partitioned into a training set (60%) to train the models, a validation set (20%) to tune hyperparameters, and a test set (20%) to independently assess model performance. Partitioning was performed in a randomly stratified manner to preserve the distribution of gender, age, and 30-day adherence status across the subsets. Each patient was exclusively associated with one of the three subsets. Missing data (1.33%) were imputed using *k*-nearest neighbors (*k* = 5).

**Fig. 1:**
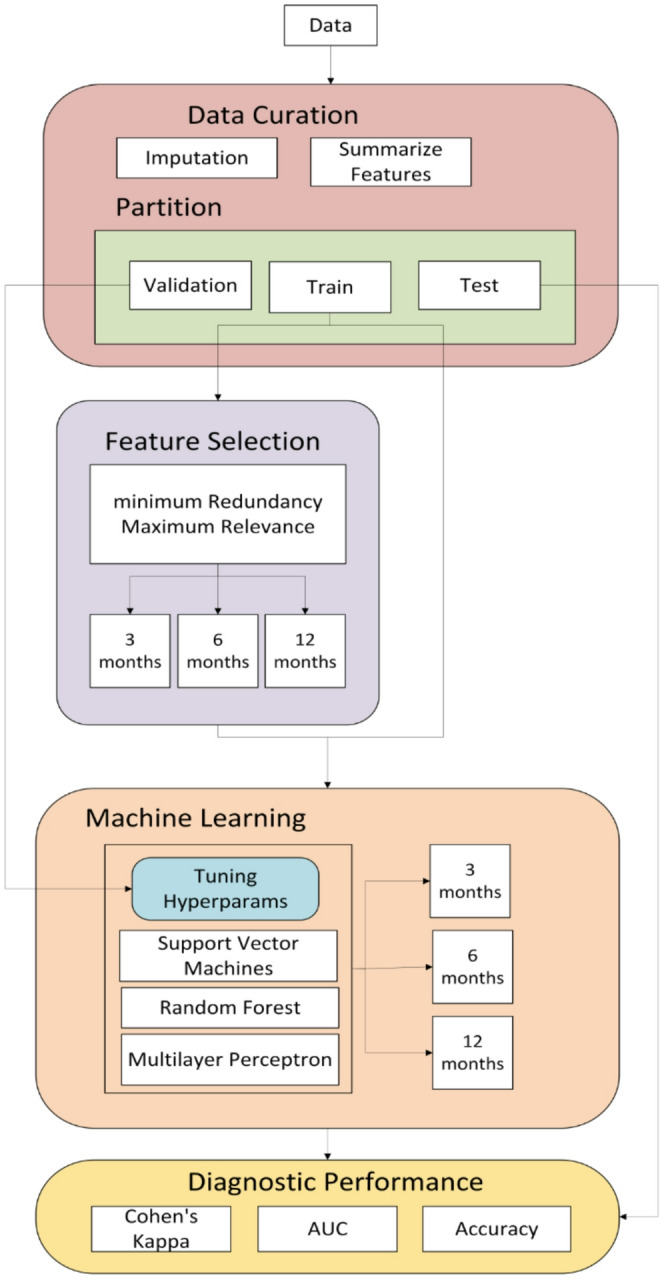
Proposed methodological workflow for defining the most relevant factors related to CPAP compliance and for predicting CPAP adherence at 3, 6, and 12 months. Data was curated and partitioned into training, validation and test datasets. The training dataset was used both to proceed with feature selection and to train the machine learning models. The validation dataset was used to optimize models’ hyperparameters. The test dataset was used to compute the performance metrics

The following subsections outline the methodological framework adopted to define the most relevant factors related to CPAP adherence and to predict CPAP compliance at 3, 6, and 12 months using ML techniques. The approach is structured into three main components: (1) feature selection, where we applied a robust mRMR-based strategy to identify the most informative variables; (2) development and optimization of predictive models using SVM, RF, and MLP algorithms; and (3) statistical analysis. The overall workflow carried out in this study is summarized in Fig. 1.

**Table 1:** Comparison between adherent and non-adherent patients at baseline (first 30 days of CPAP use)

	All	Non-Adherent (at baseline)	Adherent (at baseline)	*p*-value*
**Subjects,** *n* (%)	2180 (100%)	953 (43.72%)	1227 (56.28%)	---
**Female, n **(%)	569 (26.10%)	231 (10.60%)	338 (15.50%)	<0.01**
**Age, years**	56.5 [47, 66]	54 [44, 65]	58 [49, 66]	<0.01**
**BMI (kg/m** ^2^ **)**	29.8 [26.8, 34.0]	30.0 [26.9, 34.4]	29.7 [26.7, 33.9]	0.2328
**Active-Smoker, n **(%)	536 (24.59%)	279 (12.80%)	257 (11.79%)	0.3644
**Ex-Smoker, n** (%)	855 (39.22%)	346 (15.87%)	509 (23.35%)	<0.01**
**Non-Smoker, **n (%)	789 (36.19%)	328 (15.87%)	461 (21.15%)	<0.01**
**Hypertension, n** (%)	1211 (55.55%)	508 (23.30%)	703 (32.25%)	<0.01**
**Diabetes, n** (%)	509 (23.35%)	241 (11.06%)	268 (12.29%)	0.2491
**COPD, n **(%)	158 (7.25%)	80 (3.67%)	78 (3.58%)	0.9366
**Asthma, n** (%)	200 (9.17%)	95 (4.36%)	105 (4.82%)	0.5246
**Psychiatric disorder, n **(%)	543 (24.91%)	254 (11.65%)	289 (13.26%)	0.1445
**Cerebrovascular, n** (%)	138 (6.33%)	50 (2.29%)	88 (4.04%)	<0.01**
**Cardiovascular, n **(%)	616 (28.26%)	278 (12.75%)	338 (15.51%)	<0.01**
**CKD, n** (%)	172 (7.89%)	68 (3.12%)	104 (4.77%)	<0.01**

*p-value from the Wilcoxon rank-sum test for continuous variables and from Chi-square test for categorical variables

**p-value < 0.05

Median [Q1, Q3] was used for numerical data, count (percentage) for categorical data

BMI: body mass index; COPD: chronic obstructive pulmonary disease; CKD: chronic kidney disease

**Table 2:** OSA characterization from diagnostic PSG for adherent and non-adherent groups at baseline (first 30-days of CPAP use)

	All	Non-Adherent (at baseline)	Adherent (at baseline)	*p*-value*
**Subjects,** *n (%)*	2180 (100%)	953 (43.72%)	1227 (56.28%)	---
**TRT, min**	444 [425, 459]	443 [422, 459]	445 [428, 459]	0.0630
**TST, min**	376.5 [336.0, 408.5]	373.5 [333.5, 407.5]	378.5 [338.5, 409.5]	0.0685
**N1, **%	12.3 [6.8, 20.8]	12.4 [6.7, 20.4]	12.3 [6.9, 21.2]	0.7771
**N2,** %	50.6 [32.5, 50.6]	41.0 [33.1, 50.8]	40.1 [32.0, 50.4]	0.1722
**N3, **%	24.8 [16.1, 33.5]	24.6 [16.1, 33.5]	25.10 [16.25, 33.60]	0.6301
**REM, **%	16.4 [10.8, 22.4]	16.1 [10.8, 22.0]	16.6 [11.0, 22.7]	0.1866
**Latency**	14.0 [6.0, 28.5]	13.0 [5.5, 28.5]	14.5 [6.5, 29.0]	0.1628
**REM latency**	122.0 [83.0, 185.5]	117.5 [80.5, 184.5]	128.0 [84.5, 185.8]	0.0689
**AHI (events/h)**	47.8 [30.0, 69.6]	45.8 [28.5, 67.7]	48.9 [31.5, 71.2]	0.032**
**Moderate OSA** ^(1**)**^**, n** (%)	62 (2.84%)	32 (1.47%)	30 (1.38%)	0.8991
**Severe OSA** ^(2)^**, n **(%)	2118 (97.16%)	921 (42.25%)	1197 (54.91%)	<0.01**
**ODI3 (events/h)**	41.2 [23.6, 63.4]	39.7 [22.4, 62.4]	42.3 [24.6, 64.6]	0.0508
**CT90** (%)	13.3 [3.3, 36.1]	12 [2.7, 34.6]	14.7 [3.9, 37.5]	0.039**

*p-value from the Wilcoxon rank-sum test for continuous variables and from Chi-square test for categorical variables

**p-value < 0.05

Median [Q1, Q3] was used for numerical data, count (percentage) for categorical data

TRT: total recording time; TST: total sleep time; N1: proportion spent in non-rapid eye movement sleep stage 1; N2: proportion spent in non-rapid eye movement sleep 2; N3: proportion spent in non-rapid eye movement sleep 3; REM: proportion spent in rapid eye movement stage; REM: rapid eye movement; AHI: apnea-hypopnea index; OSA: obstructive sleep apnea; ODI3: oxygen desaturation index of 3%, i.e., rate of desaturations greater than or equal to 3% from baseline per hour of sleep; CT90: percentage of total sleep time spent with arterial oxygen saturation < 90%

^(1)^ 5≤AHI˂10 (events/h); ^(2)^ AHI≥10 (events/h)

### Feature selection

To identify the most relevant features associated with adherence at 3, 6, and 12 months, we implemented the mRMR algorithm [22]. The goal of mRMR is to select a subset of variables that are both highly informative about the target variable and minimally redundant with each other.

**Table 3:** Comparison of usage variables between adherent and non-adherent groups at first 30days follow-up

	All	Non-Adherent (at baseline)	Adherent (at baseline)	*p*-value*
**Subjects,** *n (%)*	2180 (100%)	953 (43.72%)	1227 (56.28%)	---
**Start in January, n **(%)	159 (7.29%)	57 (2.61%)	102 (4.68%)	<0.01**
**Start in February, n** (%)	240 (11.01%)	100 (4.59%)	140 (6.42%)	0.0117**
**Start in March, n** (%)	226 (10.37%)	95 (4.36%)	131 (6.01%)	0.0197**
**Start in April, n** (%)	142 (6.51%)	50 (2.29%)	92 (4.22%)	<0.01**
**Start in May, **n (%)	170 (7.80%)	70 (3.21%)	100 (4.59%)	0.0259**
**Start in June, n** (%)	199 (9.13%)	91 (4.17%)	108 (4.95%)	0.2567
**Start in July, n **(%)	198 (9.08%)	100 (4.59%)	98 (4.50%)	0.9434
**Start in August, n** (%)	164 (7.52%)	73 (3.35%)	91 (4.17%)	0.1842
**Start in September, n **(%)	106 (4.86%)	48 (2.20%)	58 (2.66%)	0.3821
**Start in October, n **(%)	203 (9.31%)	88 (4.04%)	115 (5.28%)	0.06776
**Start in November, n **(%)	219 (10.05%)	94 (4.31%)	125 (5.73%)	0.0424**
**Start in December, n** (%)	154 (7.06%)	87 (3.99%)	67 (3.07%)	0.1255
**CPAP pressure (units)**	8 [7, 8]	8 [7, 8]	8 [7, 8]	0.5502
**CPAP usage per night, h**	5.48 [2.58, 6.83]	2.03 [0.00, 4.00]	6.66 [5.91, 7.41]	<0.01**
**Days of use**	28 [21, 30]	20 [10, 26]	29 [28, 30]	<0.01**
**Days of use ≥ 4 h**	21 [10, 28]	8 [1, 15]	27 [24, 29]	<0.01**
**Average leakage per night**	0.0 [0.0, 2.4]	0.2 [0.0, 3.6]	0.0 [0.0, 2.4]	<0.01**
**P95 of leakage per night**	4.80 [0.28, 14.4]	4.80 [0.28, 15.6]	5.40 [0.28, 13.8]	0.6885
**Maximum leakage per night**	10.80 [0.76, 22.8]	8.40 [0.66, 22.20]	12.0 [1.2, 23.4]	0.0147**
**Residual AHI per night**	1.68 [0.64, 4.00]	1.4 [0.4, 3.8]	1.9 [0.8, 4.2]	<0.01**
**EPR level mode 1, n** (%)	1184 (54.31%)	511 (23.44%)	673 (30.87%)	<0.01**
**EPR level mode 2, n** (%)	241 (11.06%)	98 (4.50%)	143 (6.56%)	<0.01**
**EPR level mode 3, n** (%)	755 (34.63%)	344 (15.78%)	411 (18.85%)	0.0163**
**EPR Fulltime mode, n** (%)	887 (40.69%)	397 (18.21%)	490 (22.48%)	<0.01**
**EPR Off mode, n** (%)	1272 (58.35%)	548 (25.14%)	724 (33.21%)	<0.01**
**EPR Ramp Only mode, n** (%)	21 (0.96%)	8 (0.37%)	13 (0.6%)	0.3833

*p-value calculated from the Wilcoxon rank-sum test for continuous variables and from Chi-square test for categorical variables

**p-value < 0.05

Median [Q1, Q3] was used for numerical data, count (percentage) for categorical data

CPAP: continuous positive airway pressure; SD: standard deviation; P95: percentile 95^;^ AHI: apnea-hypopnea index; EPR: expiratory pressure relief

The method relies on mutual information (MI), which quantifies the amount of shared information between two random variables. The MI is defined as [22]:

1 $$\:\mathrm{M}\mathrm{I}\left(X,Y\right)=\iint\:{f}_{X,Y}\left(x,y\right)\:log\:\frac{{f}_{X,Y}\:\left(x,y\right)}{{f}_{X}\:\left(x\right)\:{f}_{Y}\:\left(y\right)}dxdy$$

where $\:X,\:Y$ are random variables; we denote the probability density function of $\:X,\:Y$ and joint $\:\left(X,Y\right)$ as $\:{f}_{X}$, $\:{f}_{Y}$ and $\:{f}_{X,Y}$ respectively. Specifically, $\:{X}_{i}$ is each of our predictors and $\:{Y}_{j}$ is the adherent status of the patient at 3, 6, and 12 months, respectively. MI captures both linear and non-linear dependencies, making it well suited for complex biomedical data [22]. Formally, the mRMR criteria seeks to maximize [22]:

2 $$\:\underset{{X}_{j}\in\:S}{{max}}\left[MI\left({X}_{i},Y\right)-\frac{1}{\left|S\right|}{\sum\:}_{{X}_{j}\in\:S}MI\left({X}_{i},{X}_{j}\right)\right]$$

where $\:S$ is the set of selected features. The stability of the identified predictors was ensured by applying the mRMR algorithm within a robust bootstrapping framework. We performed 10.000 bootstrap resamples and retained only those features selected in at least 50% of the iterations, thereby ensuring that the final feature subsets were consistent and independent of specific data partitions. This threshold was chosen to balance stability and relevance [23].

### Machine learning predictive models

Three supervised learning models (support vector machine, random forest, and multi-layer perceptron) were implemented to address the binary classification task of predicting longitudinal CPAP adherence at 3, 6, and 12 months. These models were selected because of their ability to capture complex, non-linear dependencies between baseline characteristics, early treatment usage, and compliance. To ensure optimal performance and mitigate the risk of overfitting, a systematic hyperparameter tuning procedure was established. Model hyperparameters were optimized using a 5-fold cross-validation grid search strategy on the validation set. The optimization process focused on maximizing the Cohen’s Kappa coefficient to ensure model reliability in the presence of class imbalance. The ranges of values explored for each model are detailed in each algorithm’s subsection. Following the identification of the best configuration, each model was re-trained on the full training set before final evaluation on the independent test set.

#### Support vector machine (SVM)

SVM is a supervised learning algorithm primarily designed for binary classification. SVM aims to find the optimal separating hyperplane between classes by maximizing the margin [17]. Kernel functions allow SVM to model non-linear boundaries by projecting data into higher-dimensional spaces. In our case, exploratory analysis suggested near-linear separability, so we employed a linear kernel [24]. Formally, given predictors $\:X$ and outcome $\:Y$, this idea can be expressed as minimize [17]:

3 $$\:\frac{1}{2}{w}^{2}+C{\sum\:}_{i=1}^{N}{{\upxi\:}}_{i}$$

subject to constraints [17]:

4 $$\:{y}_{i}\left(w\cdot\:{x}_{i}+b\right)\ge\:1-{{\upxi\:}}_{i},\:\:i=1,\dots\:,N,$$

5 $$\:{{\upxi\:}}_{i}\ge\:0,\:\:i=1,\dots\:,N$$

where $\:w$ is the weight vector to the hyperplane, $\:b$ is the intercept, $\:{\xi\:}_{i}$ are slack variables allowing classification errors, and $\:C$ is the regularization parameter controlling the balance between maximizing the margin and minimizing misclassification.

To optimize the predictive performance, we provided a range of values for parameter *C*, from 10^− 3^ to 10^3^, and we used kappa as the optimization metric.

#### Random forest (RF)

RF is an ensemble method that builds a collection of decision trees using bootstrap aggregation (bagging) and random feature selection at each split [18]. Each tree is trained on a bootstrap sample of the data, while only a random subset of predictors is considered for splitting at each node. This dual randomness reduces correlation between trees, improves generalization, and mitigates overfitting.

Formally, given predictors $\:X$ and outcome $\:Y$, RF approximates the conditional probability $\:P\left(Y|X\right)$ by averaging predictions across trees [18]:

6 $$\:\widehat{f}\left(X\right)=\frac{1}{T}{\sum\:}_{t=1}^{T}{h}_{t}\left(X\right),$$

where $\:{h}_{t}\left(X\right)$ is the prediction of the $\:t$-th tree and $\:T$ is the total number of trees.

For classification, predictions are made by majority vote across trees. The number of trees and tree depth are key hyperparameters that influence bias-variance trade-offs.

To enhance the predictive performance, we provided a grid of parameters that should be explored, using kappa as the optimization metric: number of trees varied between 1 and 500, maximum depth between 1 and 20, and the minimum number of samples required to split an internal node between 20 and 750.

#### Multilayer perceptron (MLP)

MLP is a feedforward artificial neural network composed of an input layer, one or more hidden layers, and an output layer [19]. Each neuron computes a weighted sum of its inputs followed by a non-linear activation function. Training is performed via backpropagation combined with stochastic gradient descent, minimizing a loss function. The network’s ability to approximate complex non-linear functions makes it suitable for capturing intricate relationships between features and outcomes.

Let $\:M$ be the number of features in the input pattern $\:{x}_{ki}$ and $\:N$ the number of hidden units. Denote by $\:{w}_{mn}$ the weight connecting input feature $\:m$ to hidden unit $\:n$ with bias $\:{b}_{n}$, and by $\:{w}_{nl}$ the weight connecting hidden unit $\:n$ to output $\:l$ with bias $\:{b}_{l}$. The activation functions of the hidden and output units are $\:{g}_{n}$ and $\:{g}_{l}$, respectively. The output units in our MLP architecture are represented as [19]:

7 $$\:{y}_{l}\left(x,w\right)={g}_{l}\left({\sum\:}_{n=1}^{N}\left({w}_{nl}{g}_{n}\left({\sum\:}_{m=1}^{M}{w}_{mn}{x}_{ki}+{b}_{n}\right)+{b}_{l}\right)\right)$$

Hyperparameters such as the number of layers, number of neurons per layer, learning rate, and regularization parameter strongly influence performance.

To ensure the best predictive model, we provided a grid of parameters that should be explored, using kappa as the optimization metric: number of hidden layers was set between 1 and 3, number of neurons per layer varied between 10 and 125, the strength of the regularization term (α) between 10^− 3^ and 10^5^, and initial learning rate between 10^− 3^ and 10^5^.

The decision of all parameter ranges was led on the basis of previous knowledge and guidelines about the models [25].

### Statistical analysis

All feature selection procedures and statistical analysis were implemented in R (4.1.1 version), while the development of ML algorithms was implemented in Python (3.0 version).

Continuous variables were expressed as median [interquartile range], while categorical variables were summarized as counts and percentages.

The normality of continuous variables was assessed using the Shapiro-Wilk test. As most variables did not meet the assumption of normality, non-parametric methods were preferred for statistical comparisons. Specifically, group comparisons between adherent and non-adherent patients were performed using the Wilcoxon rank-sum test for continuous variables and the Chi-squared test for categorical variables.

The algorithm’s performances were measured in terms of kappa, accuracy, and AUC. Kappa was used as the main performance metric due to its robustness compared to conventional accuracy [26]. We used the following well-known cut-off points to assess the kappa values [26]: ≤ 0 indicates no agreement, 0.01–0.20 none to slight, 0.21–0.40 fair, 0.41–0.60 moderate, 0.61–0.8 substantial, and 0.81-1.00 almost perfect agreement.

The significance level was set as 0.05 for all statistical analyses.

## Results

### Feature selection

After applying the feature selection algorithm separately for each time window (3, 6, and 12 months), we identified the most relevant characteristics associated with CPAP adherence at each timepoint.

- At 3 months: gender, active smoker, hypertension, EPR Ramp Only mode, median usage time, number of days of use, number of days with usage greater than 4 h, and adherence status at 30 days.
- At 6 months: hypertension, psychiatric disorder, median usage time, number of days of use, number of days with usage greater than 4 h, and adherence status at 30 days.
- At 12 months: active smoker, starting treatment in February, median usage time, number of days of use, number of days with usage > 4 h, and adherence status at 30 days.

**Fig. 2:**
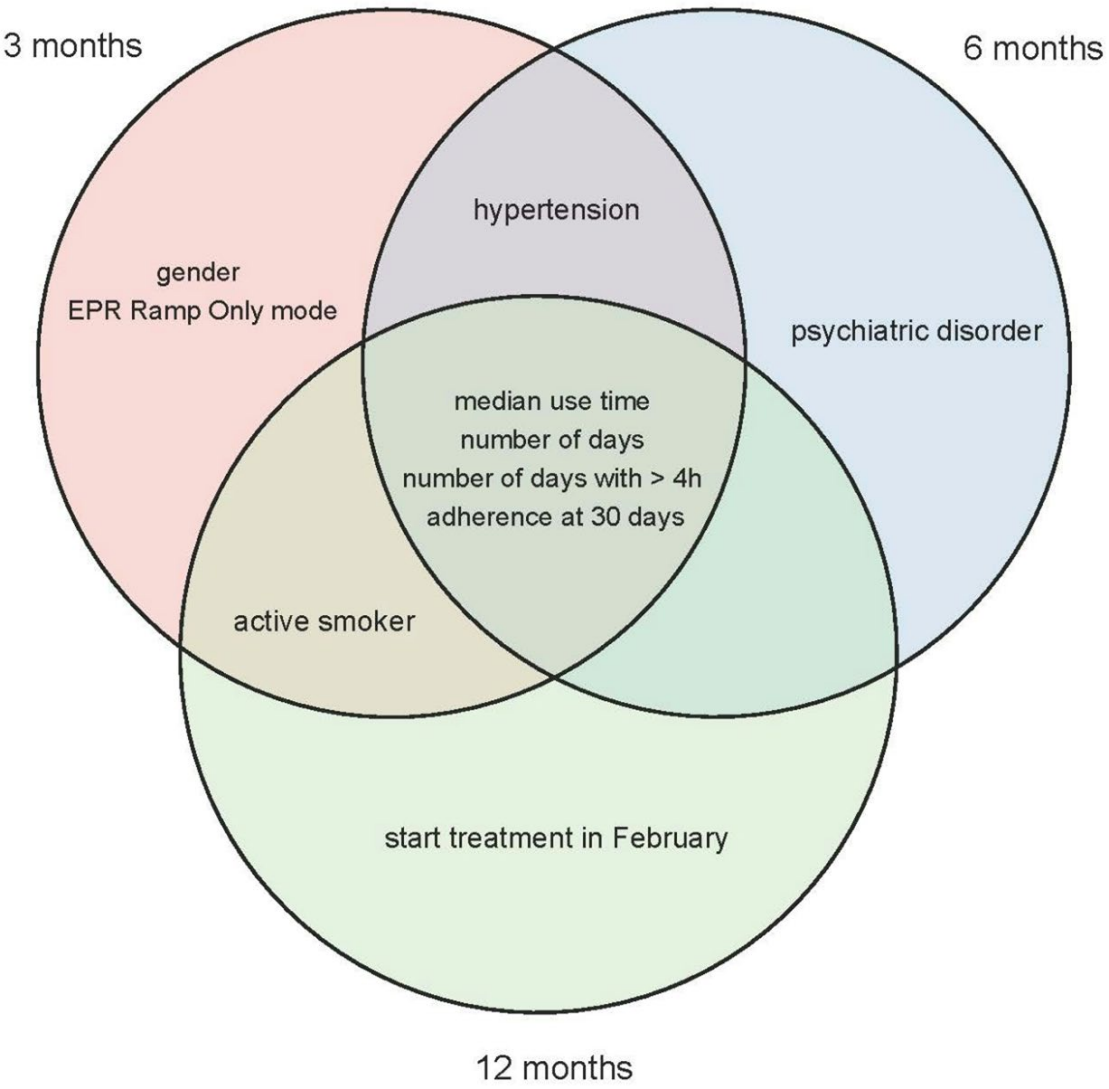
Most relevant characteristics for predicting CPAP adherence at 3, 6, and 12 months. Each circumference represents the set of features associated with its respective temporal window (3, 6 and 12 months). The different intersections represent the subsets of features shared between temporal windows

Figure 2 illustrates the overlap between selected features across the three prediction windows.

Analysis of predictor importance through MI scores derived from mRMR revealed that CPAP usage metrics from the first 30 days were the most influential features across all prediction windows. While baseline clinical and anthropometric variables exhibited lower MI values, their consistent selection in more than 50% of bootstrap iterations indicates they provide significant complementary information for predicting longitudinal adherence, as shown in Table 4.

### Machine learning predictive models

Each model (SVM with linear kernel, RF, and MLP) was trained to predict CPAP adherence at 3, 6, and 12 months. Table 5 summarizes the performance metrics across all prediction horizons.

At 3 months, the best-performing SVM used C = 0.0215, achieving a kappa of 0.801, AUC of 0.900, and accuracy of 90.1%. The RF model, with 150 estimators and maximum depth of 10, yielded kappa = 0.792, AUC = 0.896, and accuracy = 89.7%. The MLP achieved its best performance with 2 hidden layers with 20 and 10 neurons, respectively, α = 0.001, and initial learning rate = 0.0398, resulting in kappa = 0.823, AUC = 0.910, and accuracy = 91.3%.

At 6 months, SVM achieved the best performance with C = 0.0215, obtaining kappa = 0.727, AUC = 0.863, and an accuracy = 86.5%. The RF model with 25 estimators and a depth of 5 showed kappa = 0.727, AUC = 0.863, and accuracy = 86.5. The MLP, configured with 2 layers with 150 and 80 neurons, respectively, α = 3.598, and initial learning rate = 0.0077, achieved kappa = 0.718, AUC = 0.858, and accuracy = 86.0%.

At 12 months, optimal performance for SVM was found with C = 0.0215, yielding kappa = 0.670, AUC = 0.835, and accuracy = 69.8%. RF, with 150 estimators and a depth of 5, resulted in kappa = 0.698, AUC = 0.849, and accuracy = 85.1%. Lastly, MLP, using 1 hidden layer and 750 neurons, α = 0.0599, and initial learning rate = 0.4642, reported kappa = 0.661, AUC = 0.835, and accuracy = 83.1%.

**Table 4:** Mutual Information after 10,000 bootstrap resamples, expressed as mean ±1.5*standard deviation, for 3, 6, and 12 months

3 months		6 months		12 months	
Feature	Mean ± 1.5 * Standard Deviation	**Feature**	Mean ± 1.5 * Standard Deviation	**Feature**	Mean ± 1.5 * Standard Deviation
**Days of use > 4 h**	0.3695 ± 0.0457	**Days of use > 4 h**	0.2398 ± 0.1373	**Days of use > 4 h**	0.1590 ± 0.1290
**Median of use**	0.0433 ± 0.0465	**Median of use**	0.0557 ± 0.1343	**Median of use**	0.0575 ± 0.1266
**Adherence at 30 days**	0.0391 ± 0.0508	**Adherence at 30 days**	0.0312 ± 0.0264	**Adherence at 30 days**	0.0192 ± 0.0165
**Days of use**	0.0080 ± 0.0082	**Days of use**	0.0033 ± 0.0047	**Days of use**	0.0015 ± 0.0038
**Gender**	-0.0009 ± 0.0031	**Hypertension**	-0.0003 ± 0.0036	**Initiation treatment in February**	-0.0002 ± 0.0033
**Hypertension**	-0.0012 ± 0.0037	**Psychiatric disorder**	-0.0004 ± 0.0028	**Active smoker**	-0.0012 ± 0.0042
**EPR Ramp Only mode**	-0.0014 ± 0.0009				
**Active smoker**	-0.0017 ± 0.0035				

**Table 5:** Machine learning performance’s summary

Algorithm	Temporal Window	Cohen’s Kappa	AUC	Accuracy (%)	Sensitivity (%)	Specificity (%)	PPV (%)	NPV (%)	LR+	LR-
**SVM**	3 months	0.801	0.900	90.1	88.2	91.7	90.6	89.6	7.781	0.094
	6 months	0.727	0.863	86.5	83.4	89.1	86.5	0. 86.5	5.373	0.131
	12 months	0.670	0.835	69.8	81.3	85.7	85.3	81.8	4.593	0.176
**RF**	3 months	0.792	0.896	89.7	88.2	91.7	90.5	88.7	7.711	0.103
	6 months	0.727	0.863	86.5	83.4	91.2	86.5	86.5	5.373	0.131
	12 months	0.698	0.849	85.1	83.4	87.3	86.5	83.3	5.081	0.158
**MLP**	3 months	0.823	0.910	91.3	87.7	94.2	60.6	89.1	1.240	0.098
	6 months	0.718	0.858	86.0	82.9	89.1	86.1	85.9	5.188	0.137
	12 months	0.661	0.835	83.1	85.0	86.1	88.6	77.4	6.171	0.231

PPV: positive predictive value; NPV: negative predictive value; LR+: positive likelihood ratio, LR-: negative likelihood ratio; SVM: support vector machine; RF: random forest; MLP: multilayer perceptron; AUC: area under ROC curve

**Fig. 3:**
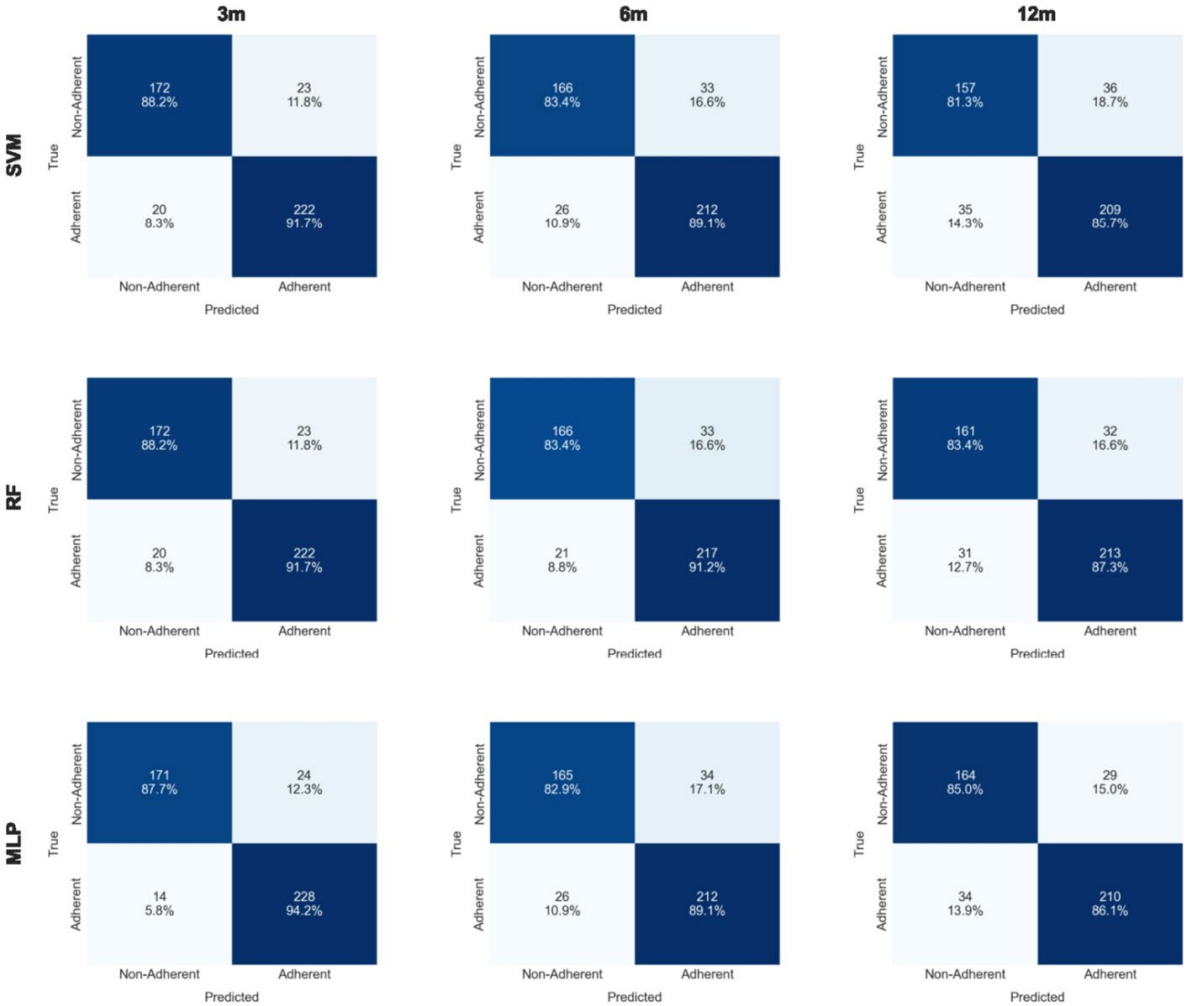
Confusion matrices of adherence prediction models at 3, 6, and 12 months. Performance is compared across three classifiers: Support Vector Machine (top row), Random Forest (middle row), and Multilayer Perceptron (bottom row). Each matrix displays the number and percentage of true positives, true negatives, false positives, and false negatives

Figure 3 presents the confusion matrices for all predictive algorithms across the three timepoints, offering insight into each model’s classification behavior and comparative strengths.

## Discussion

This study presents a novel approach to address the challenge of CPAP adherence by predicting treatment compliance at short (3 months), medium (6 months), and long term (12 months). We identified the most relevant features associated with adherence at each temporal window and implemented three ML algorithms within a reproducible and comparable framework. To the best of our knowledge, this is the first study that systematically compares the performance of multiple ML models across three distinct prediction horizons using a standardized and robust methodology. Our feature selection strategy, based on the mRMR algorithm with extensive bootstrapping, enabled the identification of key predictive factors at each stage. The findings highlight the robustness and predictive efficacy of the proposed models, exhibiting enhanced performance over existing methods reported in prior studies.

### Feature relevance across prediction horizons

By allowing different sets of features for each predictive time window, rather than enforcing a universal feature set, the approach adapts to the distinct dynamics of adherence over time. This flexibility enhances predictive performance and reflects that the determinants of CPAP adherence may vary at different stages of treatment.

Despite these differences, we identified a core subset of features consistently selected across all time points. These included the median CPAP usage time, number of days of use, number of days with usage over 4 h, and 30-day adherence status. This subset represents a measure of initial treatment engagement and device reliability, reinforcing the widely accepted view that early CPAP use is a strong predictor of long-term adherence.

Beyond the shared subset, each predictive window included distinct features. At 3 months, gender and the use of EPR Ramp Only mode were selected. The relevance of gender has been previously pointed out. Scioscia et al. [9] reported a link between gender and higher adherence in their 12-month model, while the importance of EPR mode might reflect underlying device settings or individual preferences, though it should be interpreted cautiously given its low prevalence (0.96%) in our sample. However, the inclusion of low-prevalence features was justified based on their consistent selection across 10.000 bootstrap resamples, so that only those features selected in at least 50% of the resamples were considered. Following the framework of Guyon et al. [27], these features were retained because they demonstrated high relevance to the target and provided specific complementary information that was not redundant with more frequent predictors.

At 6 months, the presence of psychiatric disorders emerged as a key predictor. This is consistent with findings from Wickwire et al. [7], who emphasized the bidirectional relationship between psychiatric conditions and sleep disturbances. These comorbidities may affect treatment motivation, tolerance, or perceived benefit, thereby influencing adherence.

For 12-month prediction, the month in which treatment was initiated (specifically, February) was found to be an influential variable. The emergence of the treatment initiation month as a long-term predictor suggests a seasonal influence on CPAP adherence. Although this relationship is novel in the context of OSA, seasonal and climatic variations are known to significantly impact symptoms and health status in other respiratory pathologies such as chronic obstructive pulmonary disease (COPD) and asthma [28, 29]. These environmental factors may influence the early adaptation period, thereby affecting the longitudinal trajectory of treatment engagement.

Certain variables were shared between two of the three predictive models. Notably, active smoking status was relevant at 3- and 12-months models, and hypertension was selected in 3- and 6-months models. These partially overlapping selections highlight that the impact of some variables on adherence may be non-linear or time dependent. For instance, the influence of hypertension may be more pronounced in the short- to mid-term, whereas smoking could affect both early discontinuation and long-term compliance.

### Machine learning algorithms’ performances

This study compares three of the most used ML algorithms (SVM, RF and MLP) under a standardized, reproducible framework to evaluate their capacity to predict CPAP adherence. For each prediction window, and using their particular optimum feature subsets, we trained and validated models based on these algorithms. The choice of these three models reflects their prominence in the current state-of-the-art [10–12] on predictive modeling for OSA-related outcomes.

To our knowledge, only one prior study by May et al. [10] attempted to compare different ML algorithms in this context. However, direct comparison with our findings is not feasible due to their use of a continuous definition of CPAP adherence, whereas our study employs a binary classification approach aligned with established clinical guidelines.

The decision to predict adherence at 3, 6, and 12 months was guided by the goal of assessing model performance across short-, medium-, and long-term horizons. To the best of our knowledge, this is the first study that systematically compares predictive performance across multiple temporal windows using the same dataset and modeling framework. Our findings provide new insight into how predictive capacity evolves over time and highlight the feasibility of long-term adherence prediction using early treatment data.

As expected, predictive performance tends to decline as the prediction window extends further from the initial 30-day monitoring period. However, this study is the first to quantify this decline systematically and to evaluate its magnitude across different models and metrics.

From a practical standpoint, the choice of the best-performing model may depend on the specific prediction horizon being targeted. In clinical practice, patients with OSA are usually monitored over long periods, and adherence support is ongoing. Therefore, rather than prioritizing one prediction window, clinicians may benefit from having reliable models for each time point, making consistent performance across all horizons more relevant than the superiority of a single model.

From an academic or methodological perspective, we propose evaluating model superiority based on the performance under the most challenging condition. In our context, this corresponds to the 12-month prediction window, the farthest point from the initial 30-day monitoring period.

In the short term, all models showed strong predictive performance. These results confirm that early CPAP usage data, combined with clinical and demographic information, can be highly informative for short-term adherence prediction.

In the medium term, RF and SVM achieved the highest performance, with a kappa of 0.727 (substantial agreement), an AUC of 0.863, and an accuracy of 86.5%. These results suggest that RF and SVM may capture subtle nonlinearities in adherence behavior better than MLP as the prediction window increases.

In the long term (12 months), RF demonstrated the most robust performance, achieving a kappa of 0.698 (substantial agreement), an AUC of 0.849, and an accuracy of 85.1%. SVM and MLP showed lower metrics, reflecting the expected decline in predictive accuracy with increased temporal distance. Nevertheless, RF’s consistent performance across temporal windows highlights its potential as a reliable tool for long-term adherence prediction in clinical settings.

However, SVM showed excellent predictive capacity in the short and medium term, matching or nearly matching the performance of the other models at 3 and 6 months. Nevertheless, its predictive ability declined more noticeably at 12 months, indicating limitations when generalizing to long-term adherence. This suggests that while SVM is a valuable option for early prediction, the selection of a linear kernel could restrict its capacity to model more complex or evolving patterns of adherence over time. MLP achieved the highest performance at 3 months, slightly surpassing the other models. This suggests that its capacity to learn non-linear relationships is especially useful for capturing early adherence behavior. However, like SVM, its performance declined in the longer term, likely due to overfitting or reduced generalizability from early input data alone.

In summary, SVM and MLP are promising options for short-term predictions, while RF offers the most stable and reliable performance across all timepoints, particularly when predicting long-term adherence.

### Comparison with previous studies

To the best of our knowledge, this is the first study to compare the performance of multiple machine learning models across different predictive time horizons using a unified dataset. Moreover, we defined the most relevant features across predictive windows. This methodological consistency enables us to trace the evolution of CPAP adherence over time while preserving clinical applicability. In practice, this means that clinicians can anticipate short-, medium-, and long-term adherence outcomes based solely on data collected during the initial 30 days of treatment.

Regarding feature selection, Scioscia et al. [9] focused on 12 month adherence and applied a combination of Fast Correlation-Based Filter (FCBF) and ReliefF algorithms. They identified variables such as age, gender, smoking status, apnea-hypopnea index, oxygen saturation during nights, time below 90% oxygen saturation (CT90), and maximum air leakage from CPAP. Eguchi et al. [11], targeting 3 month adherence, employed logistic regression followed by a learn-to-rank (LTR) algorithm and found that average daily usage, total usage duration, variability in mask pressure and usage time, average daily AHI, and average air leakage during the first week of treatment were the most relevant.

Our findings align partially with those of previous studies. In particular, the relevance of CPAP usage metrics is consistent with the results reported by Eguchi et al. [11] Additionally, several features identified by Scioscia et al. [9], such as gender and smoking status, also appeared within our selected sets, reinforcing the clinical validity of our approach.

We also compare our model performance with that reported in previous studies to assess the generalizability and clinical utility of our approach. Table 6 summarizes the performance metrics of previous studies that reported sufficient information to enable comparison. We only included studies focused on CPAP adherence prediction, although the use of AI in the context of CPAP adherence is extensive. While these studies often focused on different prediction horizons or employed different definitions of adherence, our approach allows for a meaningful comparison thanks to the methodological rigor and standardized evaluation criteria applied across models.

Among performance metrics, the AUC is the most reported in literature. Direct comparison with previous studies is challenging due to variations in prediction windows, datasets, and methodologies. However, our multi-target strategy enables meaningful alignment across time points. For instance, Scioscia et al. [9] reported an AUC of 0.729 using SVM to predict 12 month CPAP adherence in a small cohort (*n* = 86), while our models achieved higher performance at the same horizon: 0.835 (SVM), 0.849 (RF), and 0.835 (MLP). Eguchi et al. [11], using logistic regression (LR) to predict adherence at 3 months, reported an AUC of 0.763, whereas our models substantially outperformed this study, with AUCs of 0.900 (SVM), 0.896 (RF), and 0.910 (MLP). Similarly, Araujo et al. [12] evaluated several models for predicting 5-month adherence, reporting their best AUC of 0.76 using RF and LR. Our closest comparable window (6 months) yielded AUCs of 0.863 (SVM), 0.863 (RF), and 0.858 (MLP).

**Table 6:** Comparison of our proposal and previous studies

Study	*N*	Model	Prediction	Cohen’s kappa	AUC	Accuracy
**Scioscia et al. (2022)** [9]	86	SVM	12 months	-	0.729	69.5
		MLP	12 months	-	0.629	58.8
**Eguchi et al. (2022)** [11]	354	LR	3 months	-	0.763	87.2
**Araujo et al. (2021)** [12]	3785	RF	5 months	-	0.760	-
		SVM	5 months	-	0.750	-
		LR	5 months	-	0.760	-
**Our proposal**	2180	SVM	3 months	0.801	0.900	90.1
			6 months	0.727	0.863	86.5
			12 months	0.670	0.835	69.8
		RF	3 months	0.792	0.896	89.7
			6 months	0.727	0.863	86.5
			12 months	0.698	0.849	85.1
		MLP	3 months	0.823	0.910	91.3
			6 months	0.718	0.858	86.0
			12 months	0.661	0.835	83.1

SVM: support vector machine; RF: random forest; MLP: multilayer perceptron; LR: logistic regression; AUC: area under ROC curve

These findings not only demonstrate the robustness and consistency of our models across different prediction horizons but also suggest that the heterogeneity and size of our dataset (*n* = 2180) may enhance model generalizability. The combination of methodological rigor, feature selection, and balanced cohort likely contributes to improving predictive performance. Furthermore, the variability in model performance across different studies underscores the importance of dataset diversity and reinforces the clinical relevance of building generalizable models.

### Limitations and future work

Several limitations of this study should be acknowledged. First, the models were developed using retrospective data, which may introduce selection bias and limit the ability to draw causal inferences. Additionally, although model performance was assessed using internal validation, no external validation was possible due to the lack of publicly available databases with CPAP adherence data under comparable clinical conditions. To address these limitations, we plan to construct a prospective database aligned with the structure of our retrospective dataset.

Additionally, while this study focused on baseline clinical data and early usage metrics, emerging physiological markers, such as hypoxic burden and delta heart rate have recently shown potential in predicting OSA outcomes [30]. Future research will explore the integration of these novel metrics to further enhance the predictive accuracy and clinical utility of our longitudinal models.

Further limitation is related to the interpretability of the ML models, particularly SVM and MLP. In clinical practice, understanding the variables that strongly influence model decisions is crucial for enhancing interpretability, fostering clinician trust, and supporting informed decision-making. While our feature selection process partially mitigates this issue, it does not fully resolve the opacity of complex model architectures. In future work, we aim to incorporate explainable artificial intelligence (XAI) techniques to quantify the contribution of individual features within the prediction process. This will facilitate model interpretability and support integration into clinical workflows.

## Conclusions

This study demonstrates the feasibility and effectiveness of using ML algorithms to predict adherence to CPAP therapy in patients with OSA at short-, medium-, and long-term, using anthropometric and clinical data, and CPAP usage during the first 30 days of treatment. By applying a robust feature selection method (mRMR with bootstrapping) and evaluating three widely used ML algorithms (SVM, RF, and MLP), we were able to accurately predict future adherent and non-adherent patients across different time horizons.

Our findings highlight the critical role of early CPAP usage metrics (particularly nightly usage duration and adherence within the first 30 days) as strong and consistent predictors of long-term adherence. Furthermore, we identified distinct sets of time-specific features associated with each prediction window, indicating that different behavioral and physiological mechanisms may underline short-, medium-, and long-term treatment engagement. Among the evaluated models, the Random Forest (RF) demonstrated the highest robustness and stability, particularly in long-term predictions, whereas the Multilayer Perceptron (MLP) exhibited superior short-term performance. These results indicate that early identification of patients at risk of poor adherence is feasible. Integrating predictive modeling into clinical workflows could enable targeted, personalized interventions, thereby enhance treatment outcomes and alleviating the overall healthcare burden.

## Data Availability

The data that support the findings of this study are available from Río Hortega University Hospital and Oxigen Salud S.A. but restrictions apply to the availability of these data, which were used under license for the current study, and so are not publicly available. Data are, however, available from the authors upon reasonable request and with permission of from Río Hortega University Hospital and Oxigen Salus S.A.
